# Supplementary material for: Reversing protonation of weakly basic drugs greatly enhances intracellular diffusion and decreases lysosomal sequestration
Source: eLife. 2024 Dec 6;13:RP97255. doi: 10.7554/eLife.97255 (PMC11623935; doi:10.7554/eLife.97255)
Supplement: Peer review history

# Editor report: eLife Assessment

**DOI:** 10.7554/eLife.97255.3.sa0

**Authors:** Felix Campelo

**Affiliations:** Institute of Photonic Sciences Spain

**Keywords:** Convincing, Valuable

This is a **valuable** study on the diffusion rates of drug molecules in human-derived cells, presenting **convincing** data indicating that their diffusion behavior depends on their charged state. It proposes that blocking drug protonation enhances diffusion and fractional recovery, suggesting improved intracellular availability of weakly basic drugs. The findings are significant for drug design and understanding the biophysical behavior of small molecules in cells.

# Referee report: Reviewer #1 (Public review)

**DOI:** 10.7554/eLife.97255.3.sa1

Summary:

The authors set out to measure the diffusion of small drug molecules inside live cells. To do this, they selected a range of fluorescent drugs, as well as some commonly used dyes, and used FRAP to quantify their diffusion. The authors find that drugs diffuse and localize within the cell in a way that is weakly correlated with their charge, with positively charged molecules displaying dramatically slower diffusion and a high degree of subcellular localization.

The study is important because it points to an important issue related to the way drugs behave inside cells beyond the simple "IC50" metric (a decidedly mesoscopic/systemic value). The authors conclude, and I agree, that their results point to nuanced effects that are governed by drug chemistry that could be optimized to make them more effective.

Strengths:

(1) The work examines an understudied aspect of drug delivery.

(2) The work uses well-established methodologies to measure diffusion in cells

(3) The work provides an extensive dataset, covering a range of chemistries that are common in small molecule drug design

(4) The authors consider several explanations as to the origin of changes in cellular diffusion

Comments on revised version:

In general, my comments were addressed, new discussions were added, and the paper has been improved significantly, which is great.

However, despite providing very clear instructions, a lot of my comments re statistical treatment were disregarded. Bar charts still do not show the repeats as individual points. Errors bars still represent SEM, which gives a wrong idea about the spread of the data. FRAP lines are still averages, and still do not show the spread of the data.

Significance assignments are done based on average and SEMs, as opposed to the full dataset. There is nothing technically wrong with this, but it generally creates an impression that things are more reproducible/rigorous/significant than they would be if the data was shown completely.

# Referee report: Reviewer #2 (Public Review)

**DOI:** 10.7554/eLife.97255.3.sa2

Summary:

Blocking a weak base compound's protonation increased intracellular diffusion and fractional recovery in the cytoplasm, which may improve the intracellular availability and distribution of weakly basic, small molecule drugs and be impactful in future drug development.

Strengths:

(1) The intracellular distribution of drugs and the chemical properties that drive their distribution are much needed in the literature. Thus, the idea behind this paper is of relevance.

(2) The study used common compounds that were relevant to others.

(3) Altering a compound's pKa value and measuring cytosolic diffusion rates certainly is inciteful on how weak base drugs and their relatively high pKa values affect distribution and pharmacokinetics. This particular experiment demonstrated relevance to drug targeting and drug development.

(4) The manuscript was fairly well written.

Comments on revised version:

After reviewing the authors' responses to my questions and concerns, they have adequately corrected the errors, added new information and data based off the reviewers suggestions that improved the manuscript. The manuscript in its current form would add quality information to a part of the literature that is lacking much needed information.

# Author comment: Author response

**DOI:** 10.7554/eLife.97255.3.sa3

**Authors:** Debabrata Dey, Shir Marciano, Anna Poryval, Andrej Grobarz, Lucie Wohlrabova, Thomas Slanina, Gideon Schreiber

**Affiliations:** Weizmann Institute of Science Rehovot Israel; Weizmann Institute of Science Rehovot Israel; Institute of Organic Chemistry and Biochemistry of the Czech Academy of Sciences Prague Czech Republic; Institute of Organic Chemistry and Biochemistry of the Czech Academy of Sciences Prague Czech Republic; Institute of Organic Chemistry and Biochemistry of the Czech Academy of Sciences Prague Czech Republic; Institute of Organic Chemistry and Biochemistry of the Czech Academy of Sciences Prague Czech Republic; Weizmann Institute of Science Rehovot Israel

The following is the authors’ response to the original reviews.

**Public Reviews:**

**Reviewer #1 (Public Review):**

Summary:

The authors set out to measure the diffusion of small drug molecules inside live cells. To do this, they selected a range of flourescent drugs, as well as some commonly used dyes, and used FRAP to quantify their diffusion. The authors find that drugs diffuse and localize within the cell in a way that is weakly correalted with their charge, with positively charged molecules displaying dramatically slower diffusion and a high degree of subcellular localization.

The study is important because it points at an important issue related to the way drugs behave inside cells beyond the simple "IC50" metric (a decidedly mesoscopic/systemic value). The authors conclude, and I agree, that their results point to nuanced effects that are governed by drug chemistry that could be optimized to make them more effective.

We are grateful to the reviewer for summarizing the work and appreciate him/her pointing out that it is high time to consider the drug aggregation and high degree of subcellular localization while optimizing to make them more effective beyond the mesoscopic value like "IC50".

Strengths:

The work examines an understudied aspect of drug delivery.

The work uses well-established methodologies to measure diffusion in cells

The work provides an extensive dataset, covering a range of chemistries that are common in small molecule drug design

The authors consider several explanations as to the origin of changes in cellular diffusion

We are grateful to the reviewer for pointing out the strengths of the manuscript.

Weaknesses:

The results are described qualitatively, despite quantitative data that can be used to infer the strength of the proposed correlations.

The statistical treatment of the data is not rigorous and not visualized according to best practices, making it difficult for readers to assess the significance of the findings.

Some important aspects of drug behavior are not discussed quantitatively, such as the cell-to-cell or subcellular variability in concentration.

It is unclear if the observed behavior of each drug in the cell actually relates to its efficacy - though this is clearly beyond the scope of this specific work.

We have addressed the weaknesses found by the reviewer (see bellow in Reviewer #1 Recommendations For The Authors). Concerning the last point, It would have been indeed very valuable to find a relation between drug's observable behavior and their efficacy, but as the reviewer indicates, it is beyond the scope of this work.

**Reviewer #2 (Public Review):**

Summary:

Blocking a weak base compound's protonation increased intracellular diffusion and fractional recovery in the cytoplasm, which may improve the intracellular availability and distribution of weakly basic, small molecule drugs and be impactful in future drug development.

We are thankful to the reviewer for summarizing our work and acknowledging that the points raised above can be impactful in future drug development.

Strengths:

(1) The intracellular distribution of drugs and the chemical properties that drive their distribution are much needed in the literature. Thus, the idea behind this paper is of relevance.

(2) The study used common compounds that were relevant to others.

(3) Altering a compound's pKa value and measuring cytosolic diffusion rates certainly is inciteful on how weak base drugs and their relatively high pKa values affect distribution and pharmacokinetics. This particular experiment demonstrated relevance to drug targeting and drug development.

(4) The manuscript was fairly well written.

We are thankful to the reviewer for pointing out the strengths of the manuscript like the intracellular distribution of drugs and properties that drive it, which are missing in the literature.

Weaknesses:

(1) Small sample sizes. 2 acids and 1 neutral compound vs 6 weak bases (Figure 1).

We fully agree with the reviewer on this point. However, the major limitation we have faced here is the small number of drug/drug-like molecules that fluorescent with sufficient high quantum yields. For this study, we initially screened 1600 drugs for their fluorescence in the visible spectrum, and penetration into cells, resulting in 16 drugs. Of those, a small number was suitable for FRAP due to low quantum yield. For some of the molecules (Mitoxantrone, Priaquine), recovery was minimal, making them challenging to study. We added this information in the materials and method section under “Selection of drugs used in this study” (p.10).

(2) A comparison between the percentage of neutral and weak base drug accumulation in lysosomes would have helped indicate weak base ion trapping. Such a comparison would have strengthened this study.

For weakly basic compounds, the ionic form and the non-ionic form of the molecules always remain in equilibrium. The direction of the equilibrium depends on the pH of the medium, which determines the major form of the drug molecules in the solution. Our examples of GSK3 inhibitor (neutral compound, pka~7.0, as predicted by Chemaxon), shows behaviour very similar to the other basic drugs (pka>8) inside the cells. As lysosome pH is about 5.0, the neutral drug also gets protonated inside the lysosomes, as the colocalization study reveals (Figure 4). We added Fig S16 C-D, where we show co-localization of three drugs within the lysosomes showing that all the three weak base drugs colocalize to acidic lysosomes from moderately to extensively. See also in p. 11 under “Confocal microscopy and FRAP Analysis section”.

(3) When cytosolic diffusion rates of compounds were measured, were the lysosomes extracted from the image using Imaris to determine a realistic cytosolic value? In real-time, lysosomes move through the cytosol at different rates. Because weak base drugs get trapped, it is likely the movement of a weak base in the lysosome being measured rather than the movement of a weak base itself throughout the cytosol. This was unclear in the methods. Please explain.

We want to thank the reviewer for pointing this out. To clarify the point, we added to the material and method section in p. 13 the following text: “When the areas of bleach were selected in the drug-treated cell cytoplasm, we avoided the lysosomes as much as possible, within the resolution limits of the confocal microscope. Lysosomes themselves were measured to move within the cytoplasm with an diffusion coefficient of 0.03-0.071 µm2 s−1 (Bandyopadhyay et al., 2014), which is much slower than the diffusion measured for even the slowest compounds using fast Line FRAP, further validating that we did not measure lysosome diffusion.” In addition, we show that in cells after Bafilomycin A1 or Na-Azide treatments the number of lysosomes was reduced drastically (Figures S8& S9, and Figure 7), while the rates of diffusion remain very slow, similar to those measured without lysosomal inhibitors.

(4) Because weak base drugs can be protonated in the cytoplasm, the authors need to elaborate on why they thought that inhibiting lysosome accumulation of weak bases would increase cytosolic diffusion rates. Ion trapping is different than "micrometers per second" in the cytosol. Moreover, treating cells with sodium azide de-acidifies lysosomes and acidifies the cytosol; thus, more protons in the cytosol means more protonation of weak base drugs. The diffusion rates were slowed down in the presence of lysosome inhibition (Figure 7), which is more fitting of the story about blocking protonation increases diffusion rates, but in this case, increasing cytosolic protonation via lysosome de-acidification agents decreases diffusion rates. Please elaborate.

We thank the reviewer for the comment. We added to the results in p. 7 (top) the following “While we selected bleach spots to be small and located outside of lysosomes, this does not assure that some of the bleached area does not include smaller lysosomes. Therefore we investigated whether inhibiting lysosomal trapping will eliminate slow diffusion of cationic drugs.” In addition, we added to the results in p. 7-8 the following: “Comparative FRAP profiles and diffusion coefficients (Figure 7B-D and 7F-H) were slow, but conversely to Bafilomycin, sodium azide treatment did cause a further reduction is rates from Dconfocal 2.4±0.1 µm2s-1 to 1.8±0.1µm2s-1 for quinacrine and from 0.6 to 0.45 µm2s-1 for the GSK3 inhibitor (Figure 7C and G). Both Bafilomycin and sodium azide treatments resulted in elimination of drug confinement in the lysosome, and the small difference in diffusion rates may be a result of the de-acidification of the lysosomes by sodium azide, which may increase the protons in the cytosol upon treatment.”

Reviewer : A discussion of the likely impact:

The manuscript certainly adds another dimension to the field of intracellular drug distribution, but the manuscript needs to be strengthened in its current form. Additional experiments need to be included, and there are clarifications in the manuscript that need to be addressed. Once these issues are resolved, then the manuscript, if the conclusions are further strengthened, is much needed and would be inciteful to drug development.

**Reviewer #1 (Recommendations For The Authors):**

Major issues:

The paper suffers from poor statistical treatment of the data. FRAP recovery curves should be shown for each repeat, overlaid by an average with SDs as errorbars or shaded regions shown. In bar plots, SEMs should be eliminated in favor of StdDevs. All datapoints should be shown for each bar in Figs. 3-8. To show differences in D_confocal appropriate statistical tests should be conducted. In addition it is unclear what an "independent repeat" is. Does this mean 30 separate imaging sessions/drug treatments/etc? Is it 30 cells on the same coverslip? Is it a combination of both? All reported errors, SD or SEM, should have a single significant digit. Guidelines and best practices for representing quantitative imaging data are all described and visualized in detail in Lord et al. JBS 2020.

We improved the statistics and added the individual progression curves and did the statistics on them as requested. See Figure S2 for individual FRAP curves of fluorescein, GSK3 inhibitor and quinacrine. Statistical analysis of the individual FRAP curves is in Figure 3B, 4B, 5B, 7C and G. For details see figures legends and material and methods p. 13 in “Determination of Dconfocal from FRAP results”. Line FRAP was done from the cells taken from different plates, treated independently (see text p. 13).

The extensive (and commendable!) dataset the authors have collected can be put to better use than what is currently done. The main text figures in the current form of the preprint are mostly descriptive and their discussion is qualitative, to the point where the author's conclusions are supported only anecdotally. Instead, I would much rather see panels that collate the entire dataset (both protein and drugs) numerically, comparing diffusion values in buffer/cytoplasm/nucleus for all drugs (Like Fig. S6, which is in my opinion the most important in the paper but for some reason relegated to the SI). In addition I would like to see correlations within the dataset, such as D_confocal vs. pKa, vs. concentration (as measured by overall fluorescence signal, see my comment below), vs. mw, or vs. specific chemical moieties (number of charges, aromatic rings, etc). Such correlations should be discussed in terms of a correlation coefficient if conclusions were to be drawn from them, and include errors if available.

We want to thank the reviewer for these suggestions. We now made new Figures 9, and S16 to compare multiple parameters. Figure 9C shows a clear relation between pKa and Dconfocal, but no relation was found between logP, MW or number of aromatic rings and Dconfocal. Fig. S3 also shows the relation between drug concentration and Dconfocal values. These data are now discussed in the discussion section in p. 9 (bottom).

The drug sequestration hypothesis and other conclusions brought forth by the authors could be further tested by looking at the concentration dependence of the drugs inside eachcell and/or its partitioning between different subcellular compartments. The concentration dependence of these drugs is discussed in a very anecdotal fashion using two concentrations - and despite some cases showing an effect no further studies were done. Drug concentrations in this experiment can vary between cells between repeats or even within a single repeat as a result of drug chemistry and delivery methods (microinjection/passive permeability). This is especially important since it is unclear what clinically-relevant concentrations are for each drug (or at least an IC50 for the cell types tested here). I would like to see a quantitative measure of concentrations as another metric to compare diffusion behavior (see my comment above as well).

And maybe one thing to consider in addition would be some discussion in the paper about what sub-cellular distributions might actually mean in the context of drug efficacy (asking for myself as well!) - a paragraph describing recent works on the topic with some references could be instructive.

We want to thank the reviewer for the suggestion. We added now Figure S3, showing the relation between fluorescence intensity in each cell (which is directly related to the concentration of the compound) and FRAP rates and percent recovery for fluorescein, GSK inhibitor and Quinacrine. The results show now relation between drug concentration and FRAP rates, and some relation towards percent recovery. These data are now discussed in the main text (p. 4 bottor and p.6) and in the discussion (p. 9, bottom).

Minor issues:

Readers could benefit from a schematic showing the line FRAP method. It is difficult to understand from the text.

We show now in Figure 2 the line-FRAP method, and discuss it in the introduction (p. 3 top).

Have the authors considered enrichment in the cell membrane? Summed intensity projections or co-labeling with membrane dyes could prove useful to identify if the membrane is enriched in fluorescence.

The microscopy slides, including the super-resolution image in Figure S15 do not show enrichment of membranes.

Cell extracts obtained by chemical lysis are problematic because they contain surfactants. This comparison might not be meaningful.

The reviewer is correct about surfactants; However, this is only for illustration to show the crowd density of the cell extracts compared to live cells.

Unclear why "Bleach size" plots are shown. They are not discussed in the main text.

We show now a bleach size plot in Figure 2, where we explain the method. We removed them from the other figures.

Some figure panels have a strange aspect ratio, causing text to look distorted.

We corrected the figure distortion in the revised manuscript.

How are the values of D_confocal in buffer compared with past literature? Should these not all be diffusion limited? BCECF - larger than many of the drugs used here - shows ~ 100 μm^2/s in buffer (Verkman TiBS 2002).

We discussed this in our previous work (Ref. 13, iscience 2022, Dey et al.) Dconfocal is a relative diffusion rate and should not be confused with single-molecule diffusion coefficients. FRAP cannot measure the diffusion of more than 100 μm^2/s in the buffer. However, when comparing apparent FRAP rates between different fluorophores, it is not quantitative due to the major implication of the bleach radius towards diffusion rates. The rate constant normalized by bleach radius^2 is the proper way to compare i.e., our Dconfocal. (Ref. JMB 2021, iScience 2022 by Dey et al.).

**Reviewer #2 (Recommendations For The Authors):**

Recommendations:

(1) Page 3 at the bottom of the Introduction states, "...sodium azide (Hiruma et al., 2007) inhibited accumulation in lysosomes, cellular diffusion...increased only slightly." However, Figure 7C, F shows a sodium azide-induced decrease in the Dconfocal cellular diffusion. Please clarify.

Thank you for pointing this out; we corrected it in the revised version, including adding statistics.

(2) Page 6 states, "Quinacrine accumulation in the lysosome was observed also immediately after micro-injection, with aggregation increasing over time. Dconfocal of 4.2{plus minus}0.2 µm2 s-1 was calculated from line-FRAP immediately after micro-injection, slowing to 2.2{plus minus}0.1 µm2 s-1 following 2 hours incubations, with fractional recoveries of 0.63 and 0.57 respectively." If lysosome sequestration does not have an effect on cytosolic diffusion rates as the manuscript concludes, why do the authors think the diffusion rate decreased here within 2 hours? A solid conclusion would strengthen the conclusions of this manuscript rather than passing over it.

Thank you for pointing this out. We added the following text to page 7: “It is notable that the Dconfocal for Quinacrine remained consistent regardless of Bafilomycin treatment, 2 hours after incubation (Fig. S9D, 2.4±0.1 µm2s-1). However, when measured immediately after injection, the diffusion coefficient was higher at 4.2 µm2s-1 (Fig. S5D). This result does not support the notion that the faster diffusion measured immediately after cellular injection relates to lysosomal aggregation, and would better support self-aggregation, or aggregation with other molecules in the cell, which increases over time. This notion is further supported by the almost complete lack in FRAP observed 24 hours after injection (Fig. S5C).”

(3) In the Results section, the subheading states, "Inhibition of lysosomal sequestration is only slightly increasing diffusion in cells", but the conclusion for bafilomycin was...Dconfocal values were not altered by Bafilomycin A1", and the conclusion for sodium azide was diffusion coefficients (Figure 7B-C and 7E-F) were not much changed for the two drugs and stayed low... similarly to what was observed with Bafilomycin." The clear question is what is the result, "slightly increased diffusion, decreased diffusion, or had no significant effect at all"? Please clarify the wording in the manuscript to accurately describe the results.

Indeed, a small difference is obsevered between the two treatments. We added now statistical significance to Fig. 7D and H and to Fig. S8 and S9. In addition, we clarified this point in the text in p.7-8: “Comparative FRAP profiles and diffusion coefficients (Figure 7B-D and 7F-H) were slow, but conversely to Bafilomycin, sodium azide treatment did cause a further reduction is rates from Dconfocal 2.4±0.1 µm2s-1 to 1.8±0.1µm2s-1 for quinacrine and from 0.6 to 0.45 µm2s-1 for the GSK3 inhibitor (Figure 7C and G). Both Bafilomycin and sodium azide treatments resulted in elimination of drug confinement in the lysosome, and the small difference in diffusion rates may be a result of the de-acidification of the lysosomes by sodium azide, which may increase the protons in the cytosol upon treatment.”

(4) In Figure 8B, why was the Dconfocal for AM-fluorescein with or without sodium azide not included here? Besides consistency, the results might demonstrate significance. Please elaborate on the occlusion of this data.

Fraction recovery after FRAP of AM-fluorescein was very low. Calculating Dconfocal rates with such low fraction recovery is meaningless, as in the time of measurement only a small fraction recovered. Therefore, we calculated Dconfocal only when fraction recovery was at least 0.5.

(5) Throughout the Results section, the ideas and experiments are of relevance, but the suggestions/conclusions at the end of each paragraph of this section seem lightly thought out. For example, as stated on Page 8, "...however, this did not contribute new information to the puzzle." For a chemistry paper, a chemical suggestion strengthens the manuscript.

We want to thank the reviewer for these suggestions. We now made new Figures 9, and S16 to compare multiple parameters. Figure 9C shows a clear relation between pKa and Dconfocal, but no relation was found between logP, MW or number of aromatic rings and Dconfocal. Fig. S16 also shows the relation between drug concentration and Dconfocal values. We revised the discussion section to giver more weith to these quantitative assessments. These data are now discussed in p. 9.

In conclusion, the manuscript's ideas are needed, but the conclusions drawn from the experiments need to be strengthened, more explanatory, and consistent with the main conclusion of the manuscript.

See answer to point 5.
